# The Anti-Acetylcholine Receptor Antibody Test in Suspected Ocular Myasthenia Gravis

**DOI:** 10.1155/2014/689792

**Published:** 2014-11-13

**Authors:** Jung Jin Lee, Kyung Min Koh, Ungsoo Samuel Kim

**Affiliations:** ^1^Department of Ophthalmology, Kim's Eye Hospital, Konyang University College of Medicine, Youngdeungpo 4th 156, Youngdeungpo-gu, Seoul 150-034, Republic of Korea; ^2^Department of Ophthalmology, Konyang University College of Medicine, Daejeon 302-718, Republic of Korea

## Abstract

*Aim*. To estimate the clinical significance of anti-acetylcholine receptor antibody (anti-AChR-Ab) levels in suspected ocular myasthenia gravis. *Methods*. In total, 144 patients complaining of fluctuating diplopia and ptosis were evaluated for serum levels of anti-acetylcholine receptor antibody and their medical charts were retrospectively reviewed. Subjects were classified into three groups: variable diplopia only, ptosis only, and both variable diplopia and ptosis. We investigated serum anti-AChR-Ab titer levels and performed thyroid autoantibody tests. *Results*. Patients' chief complaints were diplopia (*N* = 103), ptosis (*N* = 12), and their concurrence (*N* = 29). Abnormal anti-AChR-Ab was observed in 21 of 144 patients (14.1%). Between the three groups, mean age, number of seropositive patients, and mean anti-AChR-Ab level were not significantly different (*P* = 0.224, 0.073, and 0.062, resp.). Overall, 27.5% of patients had abnormal thyroid autoantibodies. *Conclusion*. The sensitivity of anti-AChR-Ab was 14.1% in suspected ocular myasthenia gravis and seropositivity in myasthenia gravis patients showed a high correlation with the presence of thyroid autoantibodies.

## 1. Introduction 

Myasthenia gravis (MG) is an autoimmune disease limited to specific organs in which autoantibodies attack nicotine-acetylcholine receptors in neuromuscular junctions. The hypofunction of these receptors leads to a neuromuscular transmission defect, which then causes hypofunction, fatigue, and inflammation of skeletal muscles and produces serum antibodies against muscle antigens [1]. The disease is characterized by voluntary muscle weakness caused by the neuromuscular transmission defect, ophthalmic abnormalities, such as ptosis and/or diplopia, occur in 40%–94% of cases in the early stages, and ocular muscles are affected in 97% of cases during the total morbidity period [2]. Fifty to sixty percent of patients present with purely ocular symptoms, which is referred to as ocular myasthenia gravis (OMG) [3, 4].

A diagnosis of MG is based on pathognomonic history, clinical characteristics (e.g., muscle fatigue unaffected by neurological defects or areflexia), and the results of necessary examinations. In cases of mild symptoms or questionable examination results, diagnosis relies more on pathognomonic history. Thus, it is difficult to diagnose patients whose medical histories have been poorly recorded. Ordinarily, the repetitive nerve stimulation test, the edrophonium test, anti-acetylcholine receptor antibody (anti-AChR-Ab) titers, and the ice test are used for MG diagnosis [5].

An abnormal increase in anti-AChR-Ab titer is observed in more than 90% of cases of MG and in 45%–65% of OMG cases [5]. In OMG cases accompanied by high titers, systemic MG is more likely to occur [6]. In addition, the anti-AChR-Ab titer test is less invasive than the repetitive nerve stimulation-electromyography or single fiber electromyography.

Several autoimmune diseases, such as Graves' disease, Hashimoto's disease, rheumatoid arthritis, and systemic lupus erythematous, occur more often in MG, while autoimmune thyroid disease occurs more frequently than other autoimmune conditions in MG patients [7]. Therefore, this study was conducted to investigate the sensitivity of anti-AChR-Ab levels in patients with fluctuating diplopia or ptosis, as well as to identify clinical patterns in suspected OMG cases. In addition, we evaluated the correlation between suspected OMG and autoimmune thyroid abnormalities.

## 2. Methods

This study is a retrospective study. We reviewed the medical charts of patients complaining of diplopia or ptosis with diurnal variation and fatigue between March 2010 and March 2013. In cases involving diplopia, only patients with variable angle strabismus during consecutive follow-ups were included. In addition, ptosis of OMG was defined as positive using the ice test. The exclusion criteria were as follows: previous diagnoses, including cranial nerve palsy and known strabismus, prior strabismus surgery history, and former medical history for diplopia. In addition, patients with generalized fatigue symptoms and signs were excluded to select patients with pure OMG. The protocol for this research study was reviewed and approved by the Institutional Review Board (IRB) of Kim's Eye Hospital, and all procedures conformed to the guidelines of the Declaration of Helsinki.

Subjects were classified into three groups: group 1, patients with variable diplopia; group 2, patients with only ptosis; and group 3, patients with both fluctuating diplopia and ptosis. We performed full ophthalmologic examinations and investigated the pattern of extraocular movement consisting of duction and version ability and ocular deviation using the prism alternate cover test.

We also investigated serum anti-AChR-binding Ab titers. Anti-AChR-binding Ab titers were analyzed using radioactive isotope-based radioimmunoassay (RIA) and titers of ≥0.2 nmol/L were regarded as abnormal. In addition, the thyroid autoantibody test was conducted because MG is known to be closely associated with autoimmune diseases, such as dysthyroid ophthalmopathy [8]. The thyroid autoantibody test was limited to anti-thyroglobulin antibodies (TGA), anti-microsomal antibodies (TPO-Ab), and thyroid stimulating hormone (TSH) receptor antibodies; in addition, patients underwent the thyroid function test (TFT), which measures T3, T4, free T4, and TSH levels.

First, we calculated the number of patients who tested positive for anti-AChR-Ab and each ophthalmologic feature using ocular movement tests, such as duction and version assessments, followed by the alternate prism cover test, and these results were compared between the three groups. Second, comparative analysis was performed on the anti-AChR-Ab titers in the three groups. Finally, thyroid gland abnormalities, including abnormal results for TFT and autoantibodies, such as thyroglobulin antibody (TGA, <115 IU/mL), TSH receptor antibody (TSH-RA, <1.5 U/L), and anti-thyroid microsomal autoantibody (TPO-Ab, <34 IU/mL), were compared between the three groups. Statistical analyses were performed using SPSS ver. 14.0 (IBM Corp., Armonk, NY, USA). One-way analysis of variance (ANOVA) test was used to analyze the statistical difference between anti-AChR-Ab titers and age between the three groups.

## 3. Results 

First, we chose 2,342 patients with ptosis and/or diplopia from the hospital database. Among these, 144 patients complaining of fluctuating diplopia and ptosis were included. The mean age of the subjects was 43.4 ± 16.6 years, and the subject population included 68 men and 76 women. Their chief complaints included diplopia (*N* = 103), ptosis (*N* = 12), or both (*N* = 29; Table 1). Abnormal anti-AChR-Ab levels (>0.2 nmol/L) were observed in 21 of 144 (14.5%) patients; this cohort included 11 men and 10 women, and their mean age was 43.3 ± 16.5 years. With respect to mean age, there were no statistically significant differences between subjects with normal anti-AChR-Ab levels (43.5 ± 16.7 years) and seropositive OMG patients (*P* = 0.481). Among the three groups, mean age, number of seropositive patients, and mean anti-AChR-Ab level were not significantly different (*P* = 0.224, 0.073, and 0.062, resp.).

**Figure 1:**
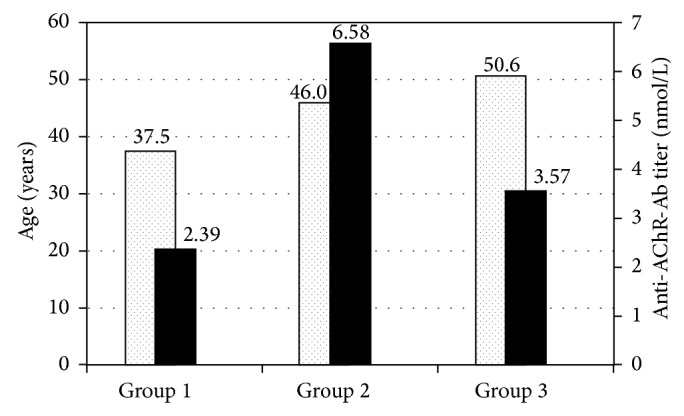
Age and anti-acetylcholine receptor antibody (anti-AChR-Ab) titer in the patients with abnormal anti-AChR-Ab.

The chief complaints of seropositive patients included diplopia, ptosis, and concurrence, which were reported by 52.4% (*N* = 11), 14.3% (*N* = 2), and 33.3% (*N* = 8) of the patients, respectively. Among groups, mean age and anti-AChR-Ab titer were not significantly different (*P* = 0.304 and *P* = 0.234, resp.; Figure 1). Seventeen of 19 patients with diplopia had strabismus: horizontal strabismus (*N* = 8), vertical strabismus (*N* = 5), concurrence (*N* = 3), and cyclotropia (*N* = 1). Of the eight patients with horizontal strabismus, five and three had exodeviation and esodeviation, respectively (Table 2); however, two patients had no definite deviation determined by alternate cover and cyclotorsion tests.

Of the total 144 subjects, 69 (59 with diplopia, 3 with ptosis, and 7 with both) patients underwent thyroid autoantibody tests. Nineteen (27.5%) of 69 patients had abnormal thyroid autoantibodies including TGA, TPO-Ab, and TSH-RA; 10 of 21 seropositive MG patients were tested for thyroid autoantibodies, and 60% of these patients had thyroid autoantibodies. Six of 19 patients tested positive for both anti-AChR-Ab and thyroid autoantibodies (Table 3). Of the 15 patients who tested positive for thyroid autoantibodies, four patients had TGA, five patients had TPO-Ab, three patients had TSR-RA, and the other seven patients tested positive for at least two of the above (Table 4).

## 4. Discussion

This study was conducted to investigate the sensitivity of anti-AChR-Ab levels in patients with fluctuating diplopia or ptosis, as well as to identify the correlation between anti-AChR-Ab levels and thyroid disorders. Abnormal anti-AChR-Ab levels were found in 14.1% (11 males and 10 females) of patients who complained of fluctuating diplopia or ptosis, 60% of whom had thyroid autoantibodies.

Our study showed a similar gender distribution to what Limburg et al. [9] reported, in that the anti-AChR-Ab titer was not related to gender. In our study, the chief complaints were diplopia (71.5%), ptosis (8.3%), and their concurrence (20.2%), whereas Mittal et al. [10] presented that the most common clinical symptom was concurrence (52% of OMG). Such discordant results could be attributed to a selection bias, because our enrolled patients were mostly recruited from a strabismus center, whereas Mittal's patients were recruited from a neuroophthalmology clinic.

In this study, 17 of 21 diplopia patients had strabismus, and vertical strabismus, including combined deviation, was found in 47.1% (8 of 17) patients. These results correspond well with those of earlier studies. For instance, Cleary et al. [11] presented that weakness of the elevator muscles, including superior rectus and inferior oblique muscles, was common in OMG, and Oosterhuis [12] suggested that the medial rectus muscle was often the first involved; however, vertical deviation was frequently found in OMG.

In the present study, there were no significant differences in mean age, anti-AChR-Ab titer, or thyroid autoantibodies between the three groups. TSH-RA is a sensitive test for Graves' disease and Hashimoto's thyroiditis, which have a high prevalence of TGA and TPO-Ab, and normal healthy controls have only a 2%–4% prevalence of thyroid autoantibodies [13]. Epidemiological studies show that autoimmune thyroid diseases occur in approximately 5%–10% of patients with MG, whereas MG is reported in a low frequency (0.2%) in patients with autoimmune thyroid diseases [14, 15]. However, Cojocaru et al. [16] reported that 40% of MA patients have thyroid autoantibodies. The present study showed that 19 (27.5%) of 69 patients had abnormal thyroid autoantibodies, including TGA, TPO-Ab, and TSH-RA. Moreover, 60% of seropositive MG patients had thyroid autoantibodies. Furthermore, patients with diplopia and ptosis simultaneously showed a high prevalence of anti-AChR-Ab and thyroid autoantibodies. The association between the two diseases seems to be attributable to autoimmune mechanisms rather than simply by chance. Recent research has shown the involvement of autoantibodies, lymphocytes, cytokines, and chemokines in the pathogenesis of MG [17]. Thus, seropositive OMG may have a higher prevalence of thyroid autoantibodies and the autoimmunity between OMG and autoimmune thyroid diseases may be significantly correlated. Subsequently, the thyroid autoantibody test could be an auxiliary test for OMG detection.

The present study has several limitations; for instance, seronegative MG patients included 85.5% of all patients and false negatives could not be defined using other diagnostic examinations, such as electromyogram, Neostigmine test, and Tensilon test. Moreover, anti-AChR-Ab levels were only detectable in about 50% of patients with OMG [18]. Therefore, some of the enrolled patients may not have MG. However, the present study focused on the sensitivity of anti-AChR-Ab as a noninvasive technique on suspected cases of MG; thus, our results provide relevant information for MG diagnoses.

## Figures and Tables

**Table 1:** The characteristics of each group.

	Diplopia (*N* = 103)	Ptosis (*N* = 12)	Diplopia and ptosis (*N* = 29)	*P* value
Male/female	57/46	5/7	17/12	0.601^*^
Mean (SD) age, yrs	42.12 ± 17.4	43.17 ± 10.5	48.17 ± 15.3	0.224^†^
Number of patients with anti-AChR-Ab^‡^ (%)	11 (10.7%)	2 (16.7%)	8 (27.6%)	0.073^*^
Mean anti-AChR-Ab^‡^ level (nmol/L)	0.26 ± 1.4	1.10 ± 2.7	0.99 ± 2.3	0.062^†^

^*^Chi-square test; ^†^one-way ANOVA; ^‡^anti-AChR-Ab: anti-acetylcholine receptor antibodies.

**Table 2:** Types of strabismus.

Strabismus	*N* = 17
Horizontal deviation	8 (47.1%)
Exodeviation	5 (29.4%)
Esodeviation	3 (17.6%)
Vertical deviation	5 (29.4%)
Combined (horizontal + vertical)	3 (17.6%)
Cyclodeviation	1 (5.9%)

**Table 3:** Thyroid autoantibodies in three groups.

	Diplopia (*N* = 59)	Ptosis (*N* = 3)	Diplopia and ptosis (*N* = 7)	*P* value
Male/female	32/27	1/2	4/3	0.763
Mean (SD) age, yrs	45.4	48.7	46.0	0.936
Hyperthyroid state	5 (8.5%)	1 (33.3%)	2 (28.6%)	0.507
Thyroid autoantibody				
Anti-thyroglobulin Ab.	9 (15.3%)	0	3 (42.9%)	0.041
Anti-microsomal Ab.	12 (20.3%)	1 (33.3%)	2 (28.6%)	0.513
TSH receptor Ab	7 (11.8%)	0	0	0.514

**Table 4:** The prevalence of anti-acetylcholine receptor antibody (anti-AChR-Ab) and thyroid autoantibodies in the three groups.

	Diplopia (*N* = 59)	Ptosis (*N* = 3)	Diplopia and ptosis (*N* = 7)	*P* value
Anti-AChR-Ab (+)	2 (3.4%)	0	0	0.043
Thyroid autoantibodies (+)	12 (20.3%)	0	1 (14.2%)	0.169
Both of them	4 (6.8%)	1 (33.3%)	3 (42.9%)	0.001
None of them	41 (69.5%)	2 (66.7%)	3 (42.9%)	0.368

## References

[1] Kuks J. B. M., Limburg P. C., Horst G., Dijksterhuis J., Oosterhuis H. J. G. H. (1993). Antibodies to skeletal muscle in myasthenia gravis. Part 1. Diagnostic value for the detection of thymoma. *Journal of the Neurological Sciences*.

[2] Ööpik M., Puksa L., Lüüs S.-M., Kaasik A.-E., Jakobsen J. (2008). Clinical and laboratory-reconfirmed myasthenia gravis: a population-based study. *European Journal of Neurology*.

[3] Benatar M., Kaminski H. J. (2007). Evidence report: the medical treatment of ocular myasthenia (an evidence-based review): report of the Quality Standards Subcommittee of the American Academy of Neurology. *Neurology*.

[4] Kupersmith M. J., Latkany R., Homel P. (2003). Development of generalized disease at 2 years in patients with ocular myasthenia gravis. *Archives of Neurology*.

[5] Benatar M. (2006). A systematic review of diagnostic studies in myasthenia gravis. *Neuromuscular Disorders*.

[6] Engel A. G. (1984). Myasthenia gravis and myasthenic syndromes. *Annals of Neurology*.

[7] Mao Z.-F., Yang L.-X., Mo X.-A. (2011). Frequency of autoimmune diseases in myasthenia gravis: a systematic review. *International Journal of Neuroscience*.

[8] Vincent A., Newsom-Davis J. (1985). Acetylcholine receptor antibody as a diagnostic test for myasthenia gravis: results in 153 validated cases and 2967 diagnostic assays. *Journal of Neurology, Neurosurgery & Psychiatry*.

[9] Limburg P. C., The T. H., Hummel Tappel E., Oosterhuis H. J. G. H. (1983). Anti-acetylcholine receptor antibodies in myasthenia gravis. Part I. Relation to clinical parameters in 250 patients. *Journal of the Neurological Sciences*.

[10] Mittal M. K., Barohn R. J., Pasnoor M., McVey A., Herbelin L., Whittaker T., Dimachkie M. (2011). Ocular myasthenia gravis in an academic neuro-ophthalmology clinic: clinical features and therapeutic response. *Journal of Clinical Neuromuscular Disease*.

[11] Cleary M., Williams G. J., Metcalfe R. A. (2008). The pattern of extra-ocular muscle involvement in ocular myasthenia. *Strabismus*.

[12] Oosterhuis H. J. G. H. (1982). The ocular signs and symptoms of myasthenia gravis. *Documenta Ophthalmologica*.

[13] Nakamura H., Usa T., Motomura M., Ichikawa T., Nakao K., Kawasaki E., Tanaka M., Ishikawa K., Eguchi K. (2008). Prevalence of interrelated autoantibodies in thyroid diseases and autoimmune disorders. *Journal of Endocrinological Investigation*.

[14] Seybold M. E. (1983). Myasthenia gravis. A clinical and basic science review. *The Journal of the American Medical Association*.

[15] Yaman A., Yaman H. (2003). Ocular myasthenia gravis coincident with thyroid ophthalmopathy. *Neurology India*.

[16] Cojocaru I. M., Cojocaru M., Muşuroi C. (2000). Study of anti-striational and anti-thyroid antibodies in patients with myasthenia gravis. *Romanian Journal of Internal Medicine*.

[17] Polymeris A., Karoutsou E., Doumouchtsis K. (2012). Seronegative myasthenia gravis and Graves' disease. Is there a link?. *Experimental and Clinical Endocrinology and Diabetes*.

[18] Kupersmith M. J., Moster M., Bhiiiyan S., Warren F., Weinberg H. (1996). Beneficial effects of corticosteroids on ocular myasthenia gravis. *Archives of Neurology*.

